# Immunotherapy as second‐line treatment and beyond for non‐small cell lung cancer in a single center of China: Outcomes, toxicities, and clinical predictive factors from a real‐world retrospective analysis

**DOI:** 10.1111/1759-7714.13488

**Published:** 2020-05-29

**Authors:** Minjiang Chen, Qiang Li, Yan Xu, Jing Zhao, Li Zhang, Lijuan Wei, Wei Zhong, Mengzhao Wang

**Affiliations:** ^1^ Department of Respiratory and Critical Care Medicine Peking Union Medical College Hospital, Chinese Academy of Medical Sciences, Peking Union Medical College Beijing China; ^2^ Department of Respiratory Beijing No.6 Hospital Beijing China

**Keywords:** Immunotherapy, non‐small cell lung cancer, real‐world study, second‐line

## Abstract

**Background:**

Real‐world evidence of second‐line treatment and beyond with immune checkpoint inhibitors (ICIs) in Chinese patients is lacking. Here, we aimed to assess the efficacy, responses, and immune‐related side effects of anti‐PD‐1 agents in real‐life practice.

**Methods:**

We retrospectively analyzed consecutive patients who received nivolumab or pembrolizumab monotherapy at Peking Medical College Hospital. We collected baseline characteristics, evaluated treatment efficacy, and categorized immune‐related adverse effects (irAEs). Predictive factors of treatment response were also determined.

**Results:**

The study included 97 patients with a median age of 64 years. The majority of patients were male, with nonsquamous histological type and advanced stage tumor, and had a history of smoking. Most patients received ICIs as second‐line therapy. Expression of PD‐L1 was detected in 34.11% patients. Overall response rate (ORR) and disease control rate (DCR) were 16.49% and 60.82%, respectively. None of the patients achieved complete response (CR). The median PFS and OS were150 days and 537 days, respectively. The incidence of immune‐related toxicities was similar to the one previously reported. Patients with driver gene mutations had shorter PFS than patients without, while patients who encountered irAE had relatively longer PFS.

**Conclusions:**

The real‐world clinical outcome of ICIs in second‐ and further‐line NSCLC therapy is promising. Several characteristics may have predictive value for efficacy. Occurrence of irAEs during treatment was acceptable and could be an independent positive predictive for PFS.

**Key points:**

**Significant findings of the study:**

- Efficacy and safety profile of ICIs as second‐line treatment or above for patients with NSCLC are promising in real world circumstances
- Incidence and median time to the occurrence of irAEs vary between organs

**What this study adds:**

- Driver gene mutations are associated with lower progression‐free survival
- Occurrence of irAEs is associated with higher progression‐free survival

## Introduction

Advances in immuno‐oncology have caused a dramatic shift in the treatment landscape of advanced non‐small cell lung cancer (NSCLC) in recent years. Immune checkpoint inhibition therapy, which has a profoundly different cure mechanism from target therapy or chemotherapy, restoring the efficacy of tumor‐specific T cells within the tumor microenvironment thereby enhance immune response and has shown promising outcomes in NSCLC.^1^ In several clinical trials, immune checkpoint inhibitor (ICI) therapies significantly improved progression‐free survival (PFS) compared with chemotherapy.^2^, ^3^, ^4^, ^5^, ^6^, ^7^ Consequently, the Food and Drug Administration (FDA) and the National Medical Products Administration (NMPA) approved two PD‐1 inhibitors, pembrolizumab and nivolumab, for the first‐ and second‐line treatment of NSCLC.

Although significant responses of NSCLC to PD‐1 inhibitors have been demonstrated in clinical trials, there is a paucity of data in real‐world. In real‐world settings, patient cohorts are more heterogeneous, and some patients are unsuitable for clinical trials. Real‐world evidence (RWE) includes data from patients of different background and can help improving management of individual patients. The aim of this study was to assess the efficacy, responses, and immune‐related side effects of anti‐PD‐1 agents in real‐life practice after the approval of anti‐PD‐1 therapy in China. We also analyzed treatment alternatives to PD‐1 inhibitors after tumor progression. To our knowledge, this study is the largest single site retrospective study of real‐world in China.

## Methods

### Study design and patient population

This study was conducted in Peking Union Medical College Hospital (PUMCH). Patients were collected from a prospective cohort data base (CAPTRA‐Lung Study).^8^ The inclusion criteria were: (i) pathologically or cytologically diagnosed with advanced or recurrent NSCLC; (ii) progressed after at least first‐line treatment with platinum‐based doublet chemotherapy; (iii) patients with driver gene mutations also received targeted therapy before initiating immunotherapy; and (iv) treated with second‐line monotherapy and beyond of pembrolizumab or nivolumab between 1 April 2017 and 31 December 2019 in PUMCH. Patients' demographic characteristics, stage of disease at the beginning of therapy, histology, treatment sequence, drug efficacy, survival data, gene mutation profile, and PD‐L1 expression of tumor cell using Daco 22C3 antibody were retrieved from the database and subsequently analyzed. All the information was retrospectively collected.

### Response and side effect assessment

During the treatment cycles, disease assessments were performed every six weeks. The Response Evaluation Criteria in Solid Tumors (RECIST) criteria v 1.1 were used to evaluate disease responses. Progression‐free survival (PFS) was calculated from the beginning of anti‐PD‐1 treatment to date of progression of disease or death. Overall survival (OS) was calculated from the beginning of anti‐PD‐1 treatment to death.

Adverse effects (AEs) with an immunological basis were defined immune‐related adverse effects (irAEs). All irAEs were classified and graded according to the National Cancer Institute Common Toxicity Criteria for Adverse Events (CTCAE; version 5.0).

### Statistical analysis

We conducted descriptive analyses on clinical and pathological variables. We compared variables that might be associated with clinical efficacy using univariate and multivariate Cox proportional hazards regression. Progression‐free survival (PFS) and overall survival (OS) data are presented as Kaplan‐Meier curves. Statistical analyses were performed with SPSS 20 and GraphPad Prism 8.0.

### Ethical statement

Every patient in the study had signed their informed consent. The retrospective analysis was approved by the ethic board of PUMCH.

## Results

### Clinicopathological characteristics of the patients

A total of 2430 patients received treatment at the lung cancer center of PUMCH from 1 April 2017 to 31 December 2019. Among these, 97 patients had received anti‐PD‐1 treatment as second‐line therapy or beyond. The majority of these patients were men (men: women ratio = 2.03:1), and their median age was 64 years.

Most patients had nonsquamous histology type (59.79%) and metastatic disease (77.32%). The most frequent metastatic site was contralateral lung, followed by bone, liver, and adrenal. Most patients had smoking history (58.76%). Checkpoint inhibitor was given a second‐line treatment in 72 patients (74.23%) and third or fourth line in 25 patients (25.77%). Nivolumab was given to the majority of patients (63.92%). The patients' characteristics are summarized in Table 1. The treatment choice for each patient is shown in Table S1.

**Table 1:** Characteristics of a cohort of 97 patients with advanced NSCLC

Variable	Total (n)	Percentage (%)
Age years, median (IQR)	64(57–69)	
≥65	48	49.48
<65	49	50.52
Gender		
Women	32	32.99
Men	65	67.01
Smoking status		
History of smoking	57	58.76
No history of smoking	40	41.24
Histology		
Nonsquamous	58	59.79
Squamous	39	40.21
Stage at diagnosis		
IIIb	22	22.68
IV	75	77.32
ECOG		
0–1	82	84.54
≥2	15	15.46
PD‐L1 expression status		
PD‐1 50%	11	11.34
PD‐L1 1–49%	23	23.71
PD‐L1 negative	32	32.99
Driver mutations		
Positive	21	21.65
Negative	53	54.64
Unknown	23	23.71
Therapeutic lines		
Second‐line	72	74.23
Third‐line and above	25	25.77
History of radiotherapy		
No	70	72.16
Yes	27	27.84
ICI drugs		
Pembrolizumab	35	36.08
Nivolumab	62	63.92

PD‐L1 expression and molecular biomarkers had been tested in most patients before initiating the immunotherapy. The tumor cell PD‐L1 expressions were assessed in 82 patients. Among these, only 65 patients had enough tumor tissue for the test. PD‐L1 expression of tumor cell was>50% in 11 patients (11.34%), ranged between one and 49% in 23 patients (23.71%), and was negative in 31 patients (31.96%) (Table 1). Predictive and prognostic biomarkers including *EGFR* mutations, ALK fusions, ROS1 fusions, MET‐14 skipping, RET rearrangement, and *KRAS* oncogene had been tested by next generation sequencing or amplification refractory mutation system PCR in 74 patients (including all the nonsquamous NSCLC). The analysis showed that 21 patients had driver gene mutations, including 15 cases (15.46%) of EGFR 19‐del or 21‐L858R mutations, three cases (3.09%) of ROS1 fusion, two cases (2.06%) of RET rearrangement, and one case (1.03%) of MET‐14skipping. *KRAS* was detected in eight patients (8.25%) (Table 1).

### Immunotherapy‐associated toxicity

None of the 97 patients had known prior history of autoimmune diseases or HIV infection. During anti‐PD‐1 treatment, four patients had infusion reaction at the first or second cycle, which presented as transient chill and fever. A total of 45 patients (46.39%) experienced irAEs. Of these, 19 patients had irAEs involving more than one organ. The organ most commonly involved was the skin, followed by endocrine system and liver.

**Figure 1:**
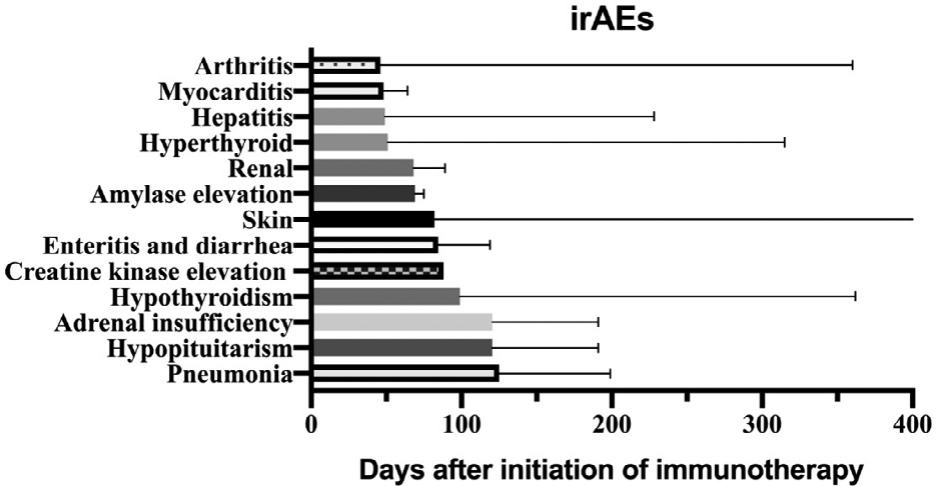
Median time from the start of immune checkpoint inhibitor (ICI) treatment to the appearance of irAEs.

The median time from immunotherapy to first irAEs was 63 days. Moreover, the median time to occurrence of irAEs varied between organs and systems (Fig 1).

Most irAEs were limited to grade 2, whereas grade 3 or 4 irAEs occurred in nine cases (9.4%). Patients were given systemic glucocorticoids for the treatment of irAEs greater than grade 3, except for endocrine irAEs, for which replacement therapies were given. Cyclosporin A, cyclophosphamide, anti‐IL‐6 antibody, and anti‐TNFα antibody were given to selected patients with critical and refractory diseases. The incidence and grades of irAEs are reported in Table 2.

**Table 2:** Immune‐related side effects of any grade during therapy

Events	No. of subjects (any grade)	Percentage (%)	No. of subjects (3–4 grade)	Percentage (%)
Cases with irAEs	45	46.39	9	9.28
Skin	25	25.77	3	3.09
Rash	17	17.53	2	2.06
Pruritus	5	5.15	0	0.00
Bulla	1	1.03	1	1.03
Psoriasis	1	1.03	0	0
Endocrine	15	15.46	3	3.09
Hyperthyroidism	8	8.24	0	0.00
Hypothyroidism	12	12.37	0	0.00
Adrenal insufficiency	2	2.06	2	2.06
Hypopituitarism	2	2.06	1	1.03
Hepatitis	8	8.24	1	1.03
Arthritis	4	4.12	0	0.00
Pneumonia	4	4.12	3	3.09
Myocarditis	3	3.09	3	3.09
Renal†	3	3.06	0	0.00
Amylase elevation	2	2.06	0	0.00
Enteritis and diarrhea	2	2.06	0	0.00
CK elevation	1	1.03	1	1.03

† Renal irAE included creatinine elevation and microscopic hematuria.

CK, creatine kinase.

Nine patients had dose interruptions, and six patients permanently stopped immunotherapy due to myocarditis (two cases), pneumonia (two cases), myocarditis plus pneumonia (one case), and grade 4 bulla (one case). Most patients experienced improvement or resolution of toxicity. Three patients died presumably as a consequence of irAEs. The causes of death were myocarditis, pneumonia, and pneumonia plus myocarditis and hepatitis.

### Response to immunotherapy and follow‐up treatments

**Figure 2:**
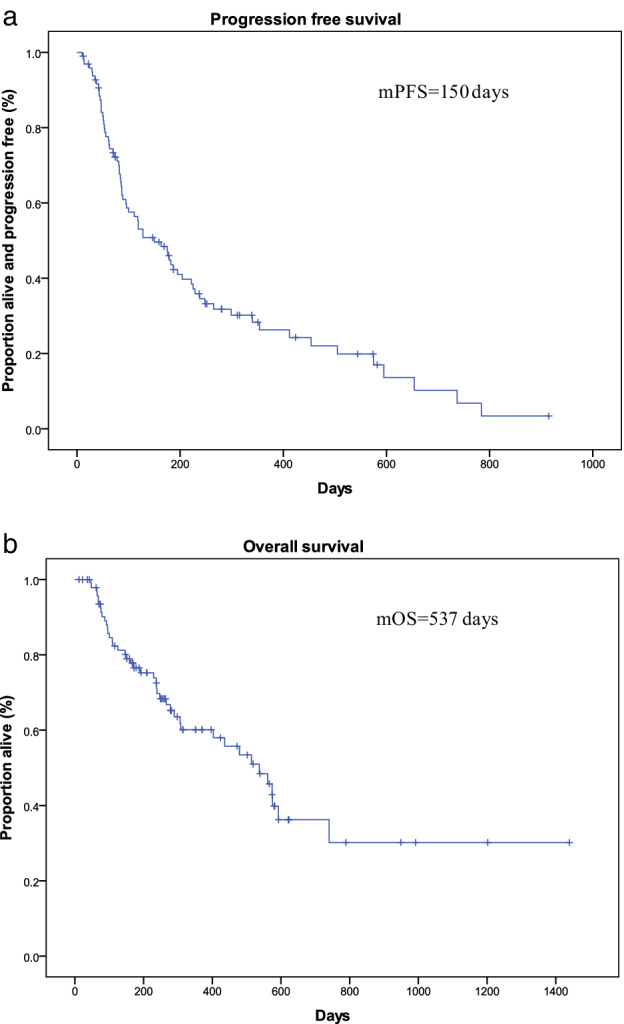
Kaplan‐Meier plot for the 97 patients. (**a**) Progression‐free survival (PFS) and (b) Overall survival (OS) from the beginning of anti‐PD‐1 treatment (m = median).

The median follow‐up time for all patients was 249 days. During this time, 72 patients (74.22%) had disease progression and 42 patients (43.30%) died. The median PFS and OS were150 days and 537 days, respectively (Fig 2). The estimated rates of OS at six months and 12 months were76.6% and 58.0%, respectively. Partial responses (PR) were achieved in 16 patients (16.49%). Stable diseases (SD) was achieved in 43 patients (44.33%). None of the patients had complete response (CR). The overall response rate (ORR) and disease control rate (DCR) were 16.93% and 60.82%, respectively.

A total of 53 patients received follow‐up therapies after progression (54.64%). The regimens included single‐drug chemotherapy (23.71%), continued immunotherapy (15.46%), and targeted therapy (15.46%). The most used mono‐chemotherapies were docetaxel (12.37%), gemcitabine (4.12%), or paclitaxel (3.09%). Targeted therapies were only used in patients who were positive to driver gene mutations, and consisted of second‐ or third‐generation tyrosine‐kinase inhibitors (TKIs) and rechallenge of first‐generation TKIs. Eight of the patients who continued immunotherapy had concurrent local treatment such as radiofrequency ablation, localized radiotherapy, and interventional embolotherapy.

### Potential clinical predictors associated with PFS during immunotherapy

**Figure 3:**
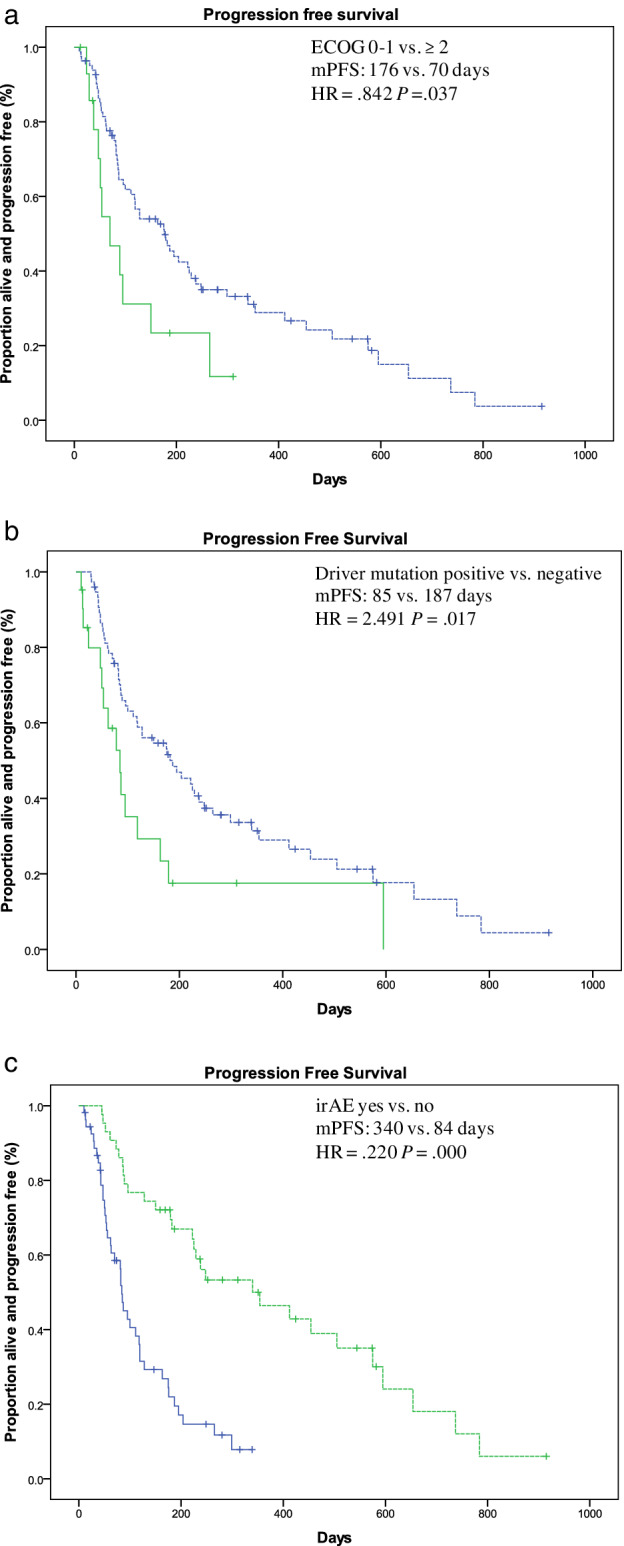
Kaplan‐Meier plot for the progression free survival (PFS) stratified by clinical factors. (**a**) ECOG score; (**b**) Driver gene mutations; and (**c**) irAE (m = median).

We further investigated the clinicopathological factors that might affect the efficacy of PD‐1 inhibitors. Univariate analysis showed that median PFS was significantly increased in patients with Eastern Cooperative Oncology Group (ECOG) performance status (PS) score 0‐1who experienced irAEs during therapy (Fig 3). Instead, PFS was significantly shorter in patients with driver gene mutations than in those without (Fig 3).The histology type, selection of different immune drugs, expression of PD‐L1, as well as other factors were not associated with PFS. Based on multivariate Cox regression analysis, having driver gene mutations was an independent negative predictive factor while occurrence of irAEs was an independent positive predictive factor of PFS (Table 3).

**Table 3:** Univariate and multivariate Cox proportional hazards regression analysis of the effect of different clinical factors on progression‐free survival

		Univariate			Multivariate	
Variable	HR	OR (95%CI)	*P*‐value	HR	OR (95%CI)	*P*‐value
Age ≥ 65 years	1.196	0.733–1.953	0.473			
Male vs. female	1.049	0.630–1.747	0.855			
Smoking history	0.813	0.506–1.307	0.813			
ECOG ≥ 2 vs.0–1	2.013	1.044–3.880	0.037	0.842	0.225–3.154	0.799
Histology squamous ‐ vs. adenocarcinoma	1.126	0.780–1.626	0.529			
Stage IIIb vs. IV	1.463	0.823–2.601	0.195	0.806	0.383–1.696	0.570
Therapy lines second vs. ≥ third	1.140	0.663–1.960	0.635			
PD‐L1 expression positive vs. negative	0.631	0.348–1.143	0.129	0.743	0.332–1.665	0.471
Previous radiation therapy	0.841	0.505–1.401	0.506			
Pembrolizumab vs. nivolumab	1.140	0.676–1.920	0.623			
Driver gene mutations	1.999	1.134–3.525	0.017	2.491	1.008–6.158	0.048
*KRAS* mutation	1.515	0.691–3.321	0.300			
Liver metastasis	1.160	0.417–3.227	0.777			
Brain metastasis	1.053	0.522–1.053	0.885			
Extra‐thorax metastasis	1.302	0.732–2.317	0.369			
irAEs	0.258	0.148–0.451	0.000	0.220	0.101–0.475	0.000

## Discussion

Patients with recurrent or advanced NSCLC for whom first‐line chemotherapy and/or targeted therapy fail generally have a poor prognosis. ICIs, which have the ability to restore the patient's antitumor immunity, are becoming the new choice for these patients. In several clinical trials, ICIs have shown a significantly higher response rate and durable clinical response than chemotherapy in patients with advanced NSCLC.^9^, ^10^, ^11^ Based on the positive results of these clinical trials, ICIs have been approved by both FDA and CFDA for the treatment of advanced NSCLC.

However, most of the evidence to date comes from clinical trials and cannot be generalized to real‐world patients. There are only a few retrospective analyses that, however, include smaller cohorts of Chinese patients.^12^, ^13^

This study retrospectively analyzed the efficacy, outcomes, side effects, and clinical factors associated with prognosis in a longitudinal cohort of real‐world patients with NSCLC receiving monotherapy of ICIs as second‐line treatment and above. To the best of our knowledge, this is one of the largest comprehensive retrospective studies of real‐world patients from mainland China who were treated with second‐line PD‐1 inhibitor monotherapy.

In published clinical trials, the ORR of second‐line ICI monotherapy ranged from 18 to 37%.^3^, ^4^The ORR in our study (16.49%) was comparable to those in previous studies, while the PFS and OS were better than those in clinical trial data (150 and 537 days, respectively).^3^, ^4^, ^14^, ^15^ This could be due to several factors. First, clinical response was evaluated by clinicians instead of independent radiology reviewers. This assessment might thus include certain biases, such as tendency to rate the patients as SD instead of progressive disease (PD), and misclassification. Therefore, DCR and PFS rates in this study could be overestimated, compared to those in studies with independent reviewers. Second, characteristics and genetic background of Chinese patients might be different from those of western patients. Third, limited cases and retrospective design of this study might affect the results.

The incidence of total irAEs, ≥ grade 3 irAE, and median time to irAEs in our study were comparable to those in previous reports.^16^ The most commonly involved organ was the skin (25.77%), but only a small proportion of skin irAEs (3.09%) were rated above grade 3, and only one case presented grade 4 bulla. All patients recovered after topical or systemic corticosteroids. Most patients continued immunotherapy except for the patient with bulla. The endocrine system was often involved during ICIs therapy, and all affected patients were successfully treated with replacement therapy and symptomatic treatment, and continued immunotherapy afterwards. Carditis and pneumonia were uncommon (4.12% and 3.09%, respectively). Systemic corticosteroids and immune suppressors were given to patients with severe heart and/or pulmonary irAEs. Most patients experienced severe clinical symptoms and/or laboratory abnormalities and had to halt ICIs permanently. Because of its low incidence and progressive course, and due to limited treatment options, immune‐related myocarditis had extremely high mortality. In our study, two patients died of myocarditis after high doses of intravenous glucocorticoids and anti‐IL‐6 antibody. In previous reports, antithymocyte globulin (ATG) was used in patients with ICI‐related severe myocarditis.^17^ However, none of the three myocarditis patients received ATG in our study. The incidence of grade 5 irAE was higher than previously reported.^18^ It might be because of the limited number of patients. Patients in this study were older and had more comorbidities and poorer performance status than the patients in clinical trials, which have been reported to be factors associated with severe irAEs^19^ might also contribute to the relatively high mortality.

Previous studies found that patients with *EGFR*‐sensitive mutations and ALK fusion did not respond well to ICI therapy.^2^, ^3^, ^15^ Preclinical studies indicate that *EGFR* mutations activate PD‐L1 expression and induce immune escape,^20^ although the underlying mechanism is unclear. In real‐world settings, cases of *EGFR* or *ALK*‐mutated patients receiving ICIs are scarce. A retrospective study showed that only one in 28 *EGFR*‐mutant or ALK‐positive patients achieved PR as best response.^21^ The benefits of ICI therapy on OS in *EGFR* or *ALK*‐mutated patients were not significant. Recently, a systemic analysis showed no benefit on OS by using second‐line nivolumab, pembrolizumab, and atezolizumab treatment in *EGFR*‐mutated patients against docetaxel.^22^ In our study, the existence of driver gene mutations was a negative predictive factor of PFS in both univariate and multivariate analysis. Nevertheless, the efficacy of ICIs in patients with driver gene mutations should be evaluated in large, prospective randomized studies in the future before any conclusion can be drawn.

IrAEs, with an incidence of 40% to 51%,^23^, ^24^, ^25^ are common side‐effects in patients receiving ICIs. Although the underlying mechanism is currently unclear, it appears that irAEs have an intimate link with antitumor effect of ICIs. In previous studies, patients who developed irAEs had significantly higher ORR as well as longer PFS than patients without.^23^, ^24^, ^25^ However, patients with severe irAEs who interrupted treatment often had a lower median OS.^26^ In our study, the incidence of, and median time to, irAEs were comparable to those in previous studies. The onset of irAEs was shown to be an independent predictive factor for PFS, in agreement with previous studies.^22^, ^23^, ^24^, ^25^ Similarly, only a small fraction of patients stopped ICI therapy due to irAEs, which is also consistent with previous studies.^25^

Furthermore, the pattern of follow‐up therapies revealed that most patients had further‐line therapies, including chemotherapy, and a few patients continued immunotherapy beyond progression. The latter group of patients had oligoprogressive diseases and had been treated with localized therapy along with maintenance ICIs. Although the therapeutic regimen might prolong the PFS2 of driver gene mutated NSCLC with CNS and/or limited systemic disease progression on targeted therapies,^27^, ^28^ its efficacy in patients of underwent ICIs needs to be further evaluated.

There are several limitations of this study. First, this is a retrospectively designed, nonrandomized study conducted in a single center. Therefore, the patient number is limited, and biases could exist in patients' inclusion criteria and efficacy evaluations. Second, most patients with *EGFR* mutations in this study had only received first‐generation TKI therapy. The efficacy of ICIs in patients resistant to third‐line TKIs still needs to be defined. Third, tumor mutation burden, which may affect the efficacy of nivolumab, was not tested in most patients. It would be interesting to collect these data for future studies. Cases from different cancer centers should also be collected and analyzed comprehensively in the future.

In conclusion, in heterogeneous real‐world settings, ICI monotherapy showed promising clinical outcomes and acceptable side effects as second‐ and further‐line treatment for patients with advanced NSCLC. Clinical factors such as driver gene mutations and appearance of irAEs were independent predictive factors for PFS. Further prospective studies are required to understand the underlying mechanism and relationship between clinical factors and ICI response in patients.

## Disclosure

The authors declare no potential conflicts of interest.

## Supporting information


**Table S1** The treatment choice of all the 97 patients.

## References

[1] Hirsch FR , Suda K , Wiens J , Bunn PA . New and emerging targeted treatments in advanced non‐small‐cell lung cancer. Lancet 2016; 388: 1012–24. 27598681 10.1016/S0140-6736(16)31473-8

[2] Reck M , Rodríguez‐Abreu D , Robinson AG *et al* Pembrolizumab versus chemotherapy for PD‐L1‐positive non‐small‐cell lung cancer. N Engl J Med 2016; 375 (19): 1823–33. 10.1056/NEJMoa1606774. 27718847

[3] Horn L , Spigel DR , Vokes EE *et al* Nivolumab versus docetaxel in previously treated patients with advanced non‐small‐cell lung cancer: Two‐year outcomes from two randomized, open‐label, phase III trials (CheckMate 017 and CheckMate 057). J Clin Oncol 2017; 35 (35): 3924–33. 10.1200/JCO.2017.74.3062. 29023213 PMC6075826

[4] Herbst RS , Baas P , Kim DW *et al* Pembrolizumab versus docetaxel for previously treated, PD‐L1‐positive, advanced non‐small‐cell lung cancer (KEYNOTE‐010): A randomised controlled trial. Lancet 2016; 387 (10027): 1540–50. 10.1016/S0140-6736(15)01281-7. 26712084

[5] Mok TSK , Wu YL , Kudaba I *et al* Pembrolizumab versus chemotherapy for previously untreated, PD‐L1‐expressing, locally advanced or metastatic non‐small‐cell lung cancer (KEYNOTE‐042): A randomised, open‐label, controlled, phase 3 trial. Lancet 2019; 393 (10183): 1819–30. 30955977 10.1016/S0140-6736(18)32409-7

[6] Gandhi L , Rodríguez‐Abreu D , Gadgeel S *et al* Pembrolizumab plus chemotherapy in metastatic non‐small‐cell lung cancer. N Engl J Med 2018; 378 (22): 2078–92. 10.1056/NEJMoa1801005. 29658856

[7] Paz‐Ares L , Luft A , Vicente D *et al* Pembrolizumab plus chemotherapy for squamous non‐small‐cell lung cancer. N Engl J Med 2018; 379 (21): 2040–51. 10.1056/NEJMoa1810865b. 30280635

[8] Xu Y , Zhang L , Fang J *et al* Establishment of a prospective multicenter cohort for advanced non‐small cell lung cancer in China (CAPTRA‐Lung study). Thorac Cancer 2018; 9 (12): 1795–800. 10.1111/1759-7714.12865. 30264504 PMC6275840

[9] Wang C , Thudium KB , Han M *et al* In vitro characterization of the anti‐PD‐1 antibody nivolumab, BMS‐936558, and in vivo toxicology in non‐human primates. Cancer Immunol Res 2014; 2 (9): 846–56. 10.1158/2326-6066.CIR-14-0040. 24872026

[10] Garon EB , Rizvi NA , Hui R *et al* Pembrolizumab for the treatment of non‐small‐cell lung cancer. N Engl J Med 2015; 372 (21): 2018–28. 10.1056/NEJMoa1501824. 25891174

[11] Gauvain C , Vauléon E , Chouaid C *et al* Intracerebral efficacy and tolerance of nivolumab in non‐small‐cell lung cancer patients with brain metastases [published correction appears in *Lung Cancer* 2019 Oct; **136**: 159]. Lung Cancer 2018; 116: 62–6. 10.1016/j.lungcan.2017.12.008. 29413052

[12] Lin SY , Yang CY , Liao BC *et al* Tumor PD‐L1 expression and clinical outcomes in advanced‐stage non‐small cell lung cancer patients treated with nivolumab or pembrolizumab: Real‐world data in Taiwan. J Cancer 2018; 9 (10): 1813–20. 10.7150/jca.24985. 29805708 PMC5968770

[13] Song P , Zhang J , Shang C , Zhang L . Real‐world evidence and clinical observations of the treatment of advanced non‐small cell lung cancer with PD‐1/PD‐L1 inhibitors. Sci Rep 2019; 9 (1): 4278 10.1038/s41598-019-40748-7. 30862891 PMC6414649

[14] Brahmer J , Reckamp KL , Baas P *et al* Nivolumab versus docetaxel in advanced squamous‐cell non‐small‐cell lung cancer. N Engl J Med 2015; 373 (2): 123–35. 10.1056/NEJMoa1504627. 26028407 PMC4681400

[15] Borghaei H , Paz‐Ares L , Horn L *et al* Nivolumab versus docetaxel in advanced nonsquamous non‐small‐cell lung cancer. N Engl J Med 2015; 373 (17): 1627–39. 10.1056/NEJMoa1507643. 26412456 PMC5705936

[16] Morita R , Okishio K , Shimizu J *et al* Real‐world effectiveness and safety of nivolumab in patients with non‐small cell lung cancer: A multicenter retrospective observational study in Japan. Lung Cancer 2020; 140: 8–18. 10.1016/j.lungcan.2019.11.014. 31838169

[17] Jain V , Mohebtash M , Rodrigo ME , Ruiz G , Atkins MB , Barac A . Autoimmune myocarditis caused by immune checkpoint inhibitors treated with antithymocyte globulin. J Immunother 2018; 41 (7): 332–5. 10.1097/CJI.0000000000000239. 29965858

[18] Wang Y , Zhou S , Yang F *et al* Treatment‐related adverse events of PD‐1 and PD‐L1 inhibitors in clinical trials: A systematic review and meta‐analysis. JAMA Oncol 2019; 5 (7): 1008–19. 10.1001/jamaoncol.2019.0393. 31021376 PMC6487913

[19] Baldini C , Martin Romano P , Voisin AL *et al* Impact of aging on immune‐related adverse events generated by anti‐programmed death (ligand)PD‐(L)1 therapies. Eur J Cancer 2020; 129: 71–9. 10.1016/j.ejca.2020.01.013. 32143106

[20] Akbay EA , Koyama S , Carretero J *et al* Activation of the PD‐1 pathway contributes to immune escape in EGFR‐driven lung tumors. Cancer Discov 2013; 3 (12): 1355–63. 10.1158/2159-8290.CD-13-0310. 24078774 PMC3864135

[21] Gainor JF , Shaw AT , Sequist LV *et al* EGFR mutations and ALK rearrangements are associated with low response rates to PD‐1 pathway blockade in non‐small cell lung cancer: A retrospective analysis. Clin Cancer Res 2016; 22 (18): 4585–93. 10.1158/1078-0432.CCR-15-3101. 27225694 PMC5026567

[22] Lee CK , Man J , Lord S *et al* Checkpoint inhibitors in metastatic EGFR‐mutated non‐small cell lung cancer‐a meta‐analysis. J Thorac Oncol 2017; 12 (2): 403–7. 10.1016/j.jtho.2016.10.007. 27765535

[23] Toi Y , Sugawara S , Kawashima Y *et al* Association of immune‐related adverse events with clinical benefit in patients with advanced non‐small‐cell lung cancer treated with nivolumab. Oncologist 2018; 23 (11): 1358–65. 10.1634/theoncologist.2017-0384. 29934411 PMC6291330

[24] Haratani K , Hayashi H , Chiba Y *et al* Association of immune‐related adverse events with nivolumab efficacy in non‐small‐cell lung cancer. JAMA Oncol 2018; 4 (3): 374–8. 10.1001/jamaoncol.2017.2925. 28975219 PMC6583041

[25] Sato K , Akamatsu H , Murakami E *et al* Correlation between immune‐related adverse events and efficacy in non‐small cell lung cancer treated with nivolumab [published correction appears in *Lung Cancer* 2018 Dec; 126: 230–231]. Lung Cancer 2018; 115: 71–4. 10.1016/j.lungcan.2017.11.019. 29290265

[26] Ksienski D , Wai ES , Croteau N *et al* Efficacy of nivolumab and pembrolizumab in patients with advanced non‐small‐cell lung cancer needing treatment interruption because of adverse events: A retrospective multicenter analysis. Clin Lung Cancer 2019; 20 (1): e97–e106. 10.1016/j.cllc.2018.09.005. 30337270

[27] Weickhardt AJ , Scheier B , Burke JM *et al* Local ablative therapy of oligoprogressive disease prolongs disease control by tyrosine kinase inhibitors in oncogene‐addicted non‐small‐cell lung cancer. J Thorac Oncol 2012; 7 (12): 1807–14. 10.1097/JTO.0b013e3182745948. 23154552 PMC3506112

[28] Tumati V , Iyengar P . The current state of oligometastatic and oligoprogressive non‐small cell lung cancer. J Thorac Dis 2018; 10 (Suppl 21): S2537–44. 10.21037/jtd.2018.07.19. 30206497 PMC6123193

