# Supplementary material for: Immunotherapy as second‐line treatment and beyond for non‐small cell lung cancer in a single center of China: Outcomes, toxicities, and clinical predictive factors from a real‐world retrospective analysis
Source: Thorac Cancer. 2020 May 29;11(7):1955–62. doi: 10.1111/1759-7714.13488 (PMC7327684; doi:10.1111/1759-7714.13488)
Supplement: Supplementary file 1 — Table S1 The treatment choice of all the 97 patients. [file TCA-11-1955-s001.docx]

Supplementary Table 1. The treatment choice of all the 97 patients

| Patient No. | Chemotherapy regiments before ICIs | Choice of ICI drugs | Patient No. | Chemotherapy regiments before ICIs | Choice of ICI drugs |
| --- | --- | --- | --- | --- | --- |
| 1 | AC | Pembrolizumab | 50 | AC+Bev | Nivolumab |
| 2 | EP | Pembrolizumab | 51 | AP | Nivolumab |
| 3 | EP，D，NI | Nivolumab | 52 | PC | Nivolumab |
| 4 | GC，A，D | Nivolumab | 53 | AC | Nivolumab |
| 5 | AP，D，G | Nivolumab | 54 | AP+Bev | Nivolumab |
| 6 | AP | Nivolumab | 55 | AC+Bev | Pembrolizumab |
| 7 | AP，D，P | Nivolumab | 56 | GC | Pembrolizumab |
| 8 | TC | Pembrolizumab | 57 | TC | Pembrolizumab |
| 9 | TC，G | Pembrolizumab | 58 | TC，A | Pembrolizumab |
| 10 | AC | Pembrolizumab | 59 | DC | Pembrolizumab |
| 11 | AC | Pembrolizumab | 60 | TC，D | Pembrolizumab |
| 12 | GP，D | Nivolumab | 61 | TC，D | Pembrolizumab |
| 13 | Ap，D，AC+Bev | Pembrolizumab | 62 | AC | Pembrolizumab |
| 14 | G | Nivolumab | 63 | AC | Pembrolizumab |
| 15 | AP，D | Pembrolizumab | 64 | TC | Pembrolizumab |
| 16 | GC | Nivolumab | 65 | TC | Nivolumab |
| 17 | EP，GC | Nivolumab | 66 | EP | Nivolumab |
| 18 | GP，D | Nivolumab | 67 | DP | Pembrolizumab |
| 19 | AP | Nivolumab | 68 | TC | Pembrolizumab |
| 20 | GP | Nivolumab | 69 | TC | Pembrolizumab |
| 21 | AC | Nivolumab | 70 | TC，G | Nivolumab |
| 22 | AC，D | Nivolumab | 71 | AC | Pembrolizumab |
| 23 | EP | Nivolumab | 72 | AC | Pembrolizumab |
| 24 | PC | Nivolumab | 73 | DC | Nivolumab |
| 25 | AP，PC+Bev | Nivolumab | 74 | TC | Pembrolizumab |
| 26 | AP | Nivolumab | 75 | EP，GP，D | Nivolumab |
| 27 | AP，D | Nivolumab | 76 | AC | Nivolumab |
| 28 | EP，DP | Nivolumab | 77 | GP，DOC，NI | Nivolumab |
| 29 | GC | Nivolumab | 78 | AP，T+A，Doc，G，S1 | Nivolumab |
| 30 | AP+A | Nivolumab | 79 | Ap | Nivolumab |
| 31 | EP | Nivolumab | 80 | AC | Pembrolizumab |
| 32 | AP+Bev | Nivolumab | 81 | AC | Nivolumab |
| 33 | AP+Bev | Nivolumab | 82 | GP，D | Pembrolizumab |
| 34 | AP | Nivolumab | 83 | AC+Bev | Pembrolizumab |
| 35 | AP | Nivolumab | 84 | Bev | Nivolumab |
| 36 | AP+Bev | Nivolumab | 85 | GP | Nivolumab |
| 37 | GP | Nivolumab | 86 | GP | Pembrolizumab |
| 38 | AP | Nivolumab | 87 | AC | Nivolumab |
| 39 | AC+Bev | Nivolumab | 88 | GP | Nivolumab |
| 40 | GP | Nivolumab | 89 | GP | Pembrolizumab |
| 41 | GP | Nivolumab | 90 | AC | Pembrolizumab |
| 42 | AC+Bev | Nivolumab | 91 | AC+Bev | Pembrolizumab |
| 43 | PC | Nivolumab | 92 | AC+A | Pembrolizumab |
| 44 | EP，GC | Nivolumab | 93 | TC | Pembrolizumab |
| 45 | AP | Nivolumab | 94 | AC | Pembrolizumab |
| 46 | PC | Nivolumab | 95 | PC | Pembrolizumab |
| 47 | AC | Nivolumab | 96 | GP，D | Nivolumab |
| 48 | AC，D | Nivolumab | 97 | AC | Nivolumab |
| 49 | AC | Nivolumab |  |  |  |

A: pemetrexed; C: carboplatin; E: etoposide; D: docetaxel, G: gemcitabine; P: cisplatin; T: paclitaxel; Bev: bevacizumab.
